# Undifferentiated spindle cell sarcoma at the anastomosis after ileocecal resection for colon cancer: A case report

**DOI:** 10.1016/j.ijscr.2024.110643

**Published:** 2024-11-25

**Authors:** Nao Kitasaki, Masatoshi Kochi, Marino Teshima, Masataka Nakagawa, Kazuhiro Toyota

**Affiliations:** Department of Gastroenterological Surgery, National Hospital Organization Higashihiroshima Medical Center, 739-0041, 513, Jike, Saijocho, Higashihiroshima, Hiroshima, Japan

**Keywords:** Primary spindle cell sarcoma, Colon cancer, Surgery, Ileocaecal resection, Case report

## Abstract

**Introduction:**

Spindle cell sarcoma (SCS) is a sarcoma subtype rarely described in the abdominal cavity, and with a worse prognosis compared with that at other sites. We report a case of SCS occurring at the anastomosis after ileal resection for colorectal cancer.

**Presentation of case:**

An 86-year-old woman with a chief complaint of abdominal pain had undergone ileal resection, D2 dissection, and hand-sewn anastomosis in 2011 to treat papillary stage I adenocarcinoma. In February 2023, the patient was referred to our hospital because of progressive anemia, and a full circumferential type 3 tumor was found at the anastomosis site after resection of the ascending colon. Biopsy revealed a malignant spindle tumor. The patient underwent an open right hemicolectomy of the colon and limited lymph node dissection. The final pathological examination revealed an undifferentiated SCS centered on the anastomosis. Postoperative follow-up imaging is ongoing, and there has been no recurrence.

**Discussion:**

SCSs are a group of tumors composed of spindle-shaped cells. Diagnosis based solely on morphology is difficult. The final diagnosis was based on the results of immunostaining and genetic testing. A diagnosis of sarcomatous transformation due to local recurrence was ruled out. Since the possibility of a dedifferentiated liposarcoma remained, a definitive diagnosis of undifferentiated SCS with an undetermined differentiation direction was not confirmed.

**Conclusion:**

SCS is a rare disease, and this is the first reported case of SCS occurring in the colon or during colonic anastomosis. Additional cases are necessary to determine an appropriate treatment strategy.

## Highlights

• Spindle cell sarcomas occurring in the intestinal tract are rare.
• We report a case at the anastomosis after ileal resection for colorectal cancer.
• The final diagnosis was based on the results of immunostaining and genetic testing.

## 1 Introduction

Primary spindle cell sarcoma (SCS) is a subtype of mesenchymal sarcoma, a broad term for cancers that arise in connective tissues. Primary mesenchymal sarcomas of the gastrointestinal system are rare, comprising just 0.1 %–3 % of all gastrointestinal tumors [1].

SCSs can occur in almost any region [2], but are more common in superficial sites such as the head, neck, and limbs [3]. Middle-aged and older adults (50–70 years) are most commonly affected, and they are more frequent in men [4]. Abdominal occurrence (133 in 3299 cases) has been reported to be an indicator of poor prognosis [2].

Various treatment options have been proposed, including chemotherapy, radiotherapy, and surgery. Surgical resection achieves a better prognosis than non-resection [3], and surgical therapy remains the gold standard for soft tissue sarcomas. However, the importance of lymph node dissection and extensive resection remains controversial.

Here we describe a case of SCS originating at the anastomosis site after ileocecal resection for ascending colon cancer. SCSs occurring in the intestinal tract are rare. To the best of our knowledge, this is the first report of an SCS occurring at an anastomosis site after colon resection for colorectal cancer. The work has been reported in line with the SCARE criteria [5].

## 2 Presentation of case

**Fig. 1:**
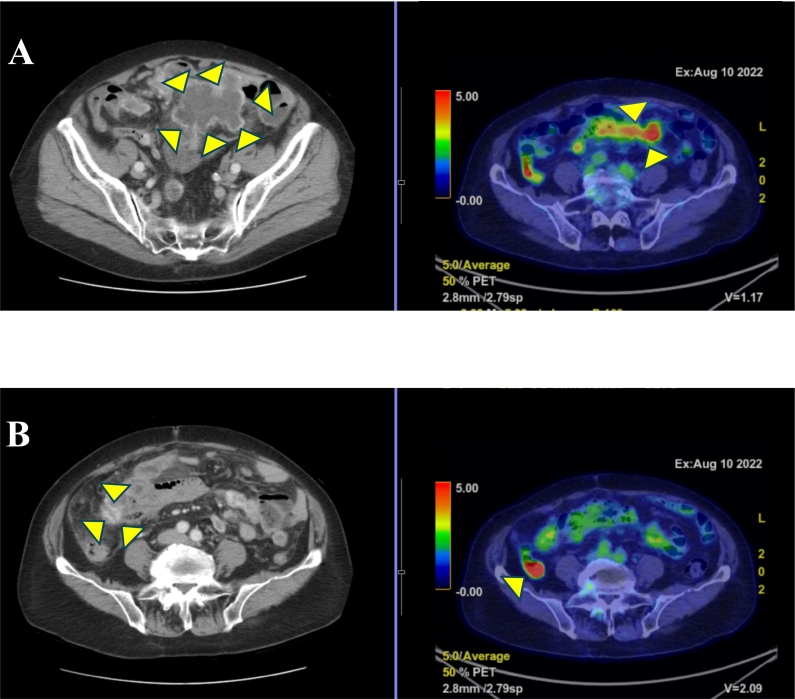
Abdominal CT and PET-CT findings. A and B: CT showed fluid accumulation with contrast effect in the pelvis and abdominal cavity. PET-CT showed significant FDG accumulation. CT, computed tomography; FDG, fluorodeoxyglucose; PET, positron emission tomography.

An 86-year-old woman presented to our hospital with a chief complaint of epigastric pain. She had undergone ileocecal resection for ascending colon cancer 10 years previously (pT2N0M0, pStageI; TNM classification 8th ed.), and no recurrence was reported. In June 2022, she visited her local doctor who referred her to our hospital after encapsulated fluid retention and scattered small nodules were found in the abdominal cavity on computed tomography (Fig. 1A,B).

### 2.1 Differential diagnosis, investigations, and treatment

**Fig. 2:**
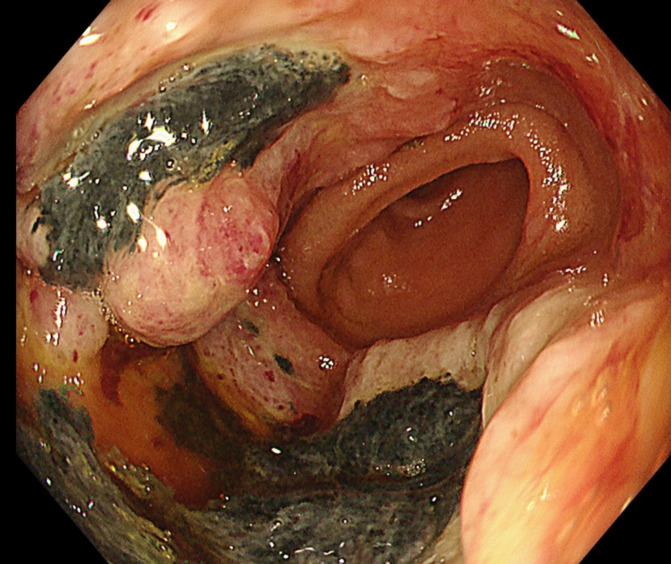
Colonoscopy findings. A circumferential type 3 tumor was found at the anastomosis after resection of the ascending colon, which had not been found 1 year before.

**Fig. 3:**
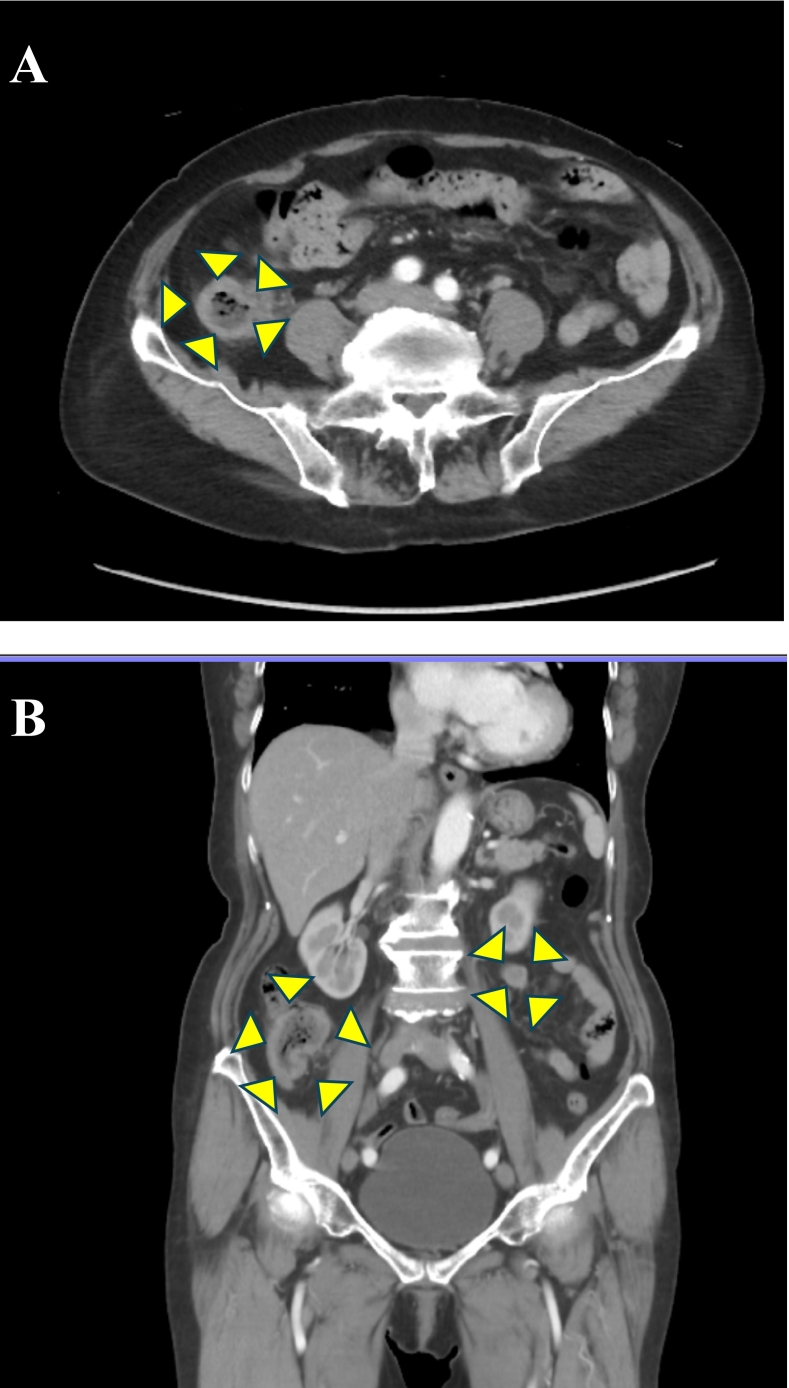
Abdominal computed tomography (CT). A and B: CT demonstrated an irregular wall thickening of 25 mm in the ascending colon and a 37 mm tumor in the mesentery of the small intestine, but no other obvious lesions.

**Fig. 4:**
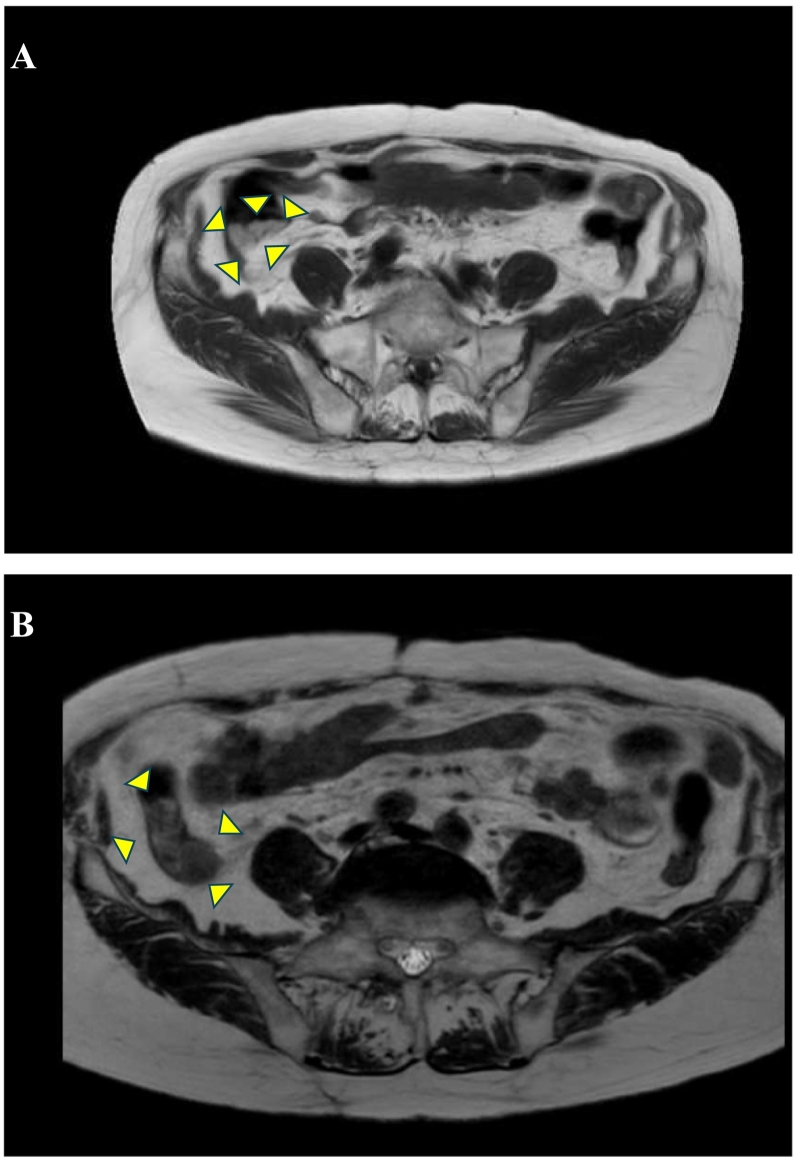
Magnetic resonance imaging (MRI) findings. MRI showed a pale high signal on T2-weighted and diffusion-weighted images, as well as a high signal on fat-suppressed T1-weighted images, suggesting mucus accumulation.

The primary lesion could not be identified, but it resolved spontaneously after approximately 6 months. The encapsulated fluid retention disappeared and no clinical cause could be identified. In February 2023, the patient was referred to our hospital for progressive anemia. A circumferential type 2 tumor was found at the anastomosis after resection of the ascending colon by colonoscopy, which had not been found in July 2022 (Fig. 2). The tumor was located at the ascending colon, and a biopsy revealed a malignant spindle tumor. Therefore, the patient was referred to our department. Blood tests showed no elevation of tumor markers, and the results of other tests were normal. Computed tomography revealed irregular wall thickening of 25 mm in the ascending colon and a 37-mm tumor in the mesentery of the small intestine, without other obvious lesions (Fig. 3A,B). Magnetic resonance imaging showed a pale high signal on T2-weighted and diffusion-weighted images, as well as a high signal on fat-suppressed T1-weighted images, suggesting mucus accumulation (Fig. 4A,B).

**Fig. 5:**
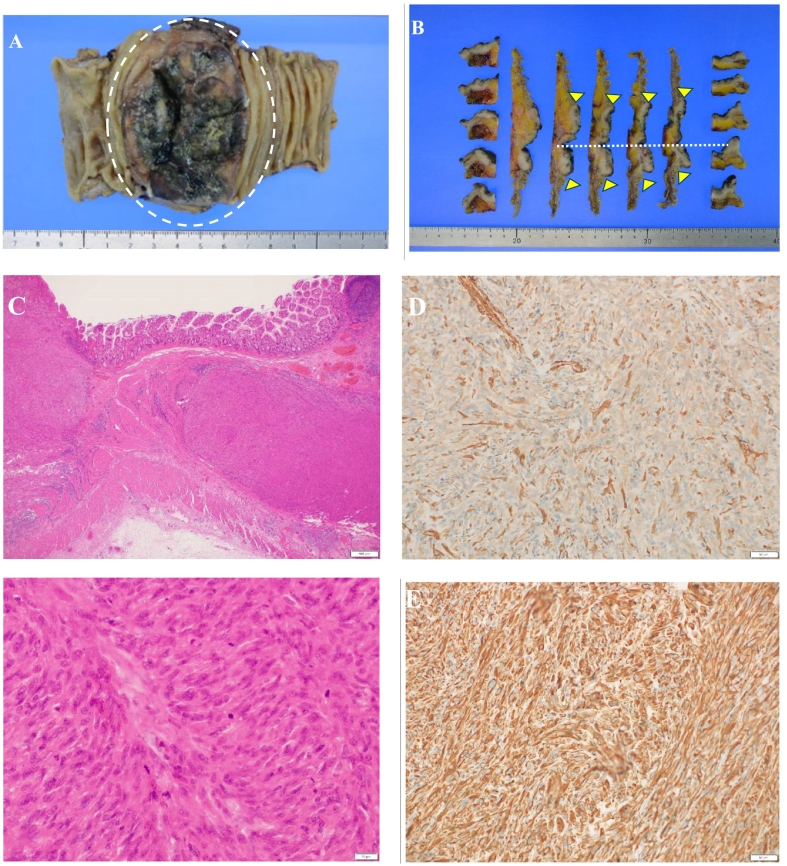
A: Macroscopically, the anastomosis around the ascending colon, which was the primary site, revealed a circumferential type 3 tumor of 84 × 52 mm in size with superficial necrotic tissue. The necrotic tissue was adhered to the superficial layer. B: The grayish-white, dense tumor was seated in the intrinsic muscularis propria, with atypical spindle-shaped cells in the tumor area, infiltrating into the subplasma membrane and showing atypical nuclear fission. The serosa layer of the colon was filled with fibrous hyperplasia, vessel hyperplasia, inflammatory cell infiltration, and necrotic tissues. The dotted line denotes the anastomosis. C and D: Further immunohistochemical analysis showed that the tumor cells were positively stained for α1-antichymotrypsin (C) and vimentin (D).

The patient was diagnosed with a malignant spindle cell tumor localized at the anastomosis. In March 2023, the patient underwent open right hemicolectomy of the colon and limited lymph node dissection (owing to previous dissection during the original surgery). Only one small nodule was found intraoperatively in the abdominal cavity, which was submitted for pathological examination and diagnosed as scar tissue. The operative time was 252 min, with a blood loss of 120 mL. The patient was discharged on postoperative day 8 without any complications. Macroscopically, the anastomosis around the ascending colon, which was the primary site, revealed a circumferential type 3 tumor sized 84 × 52 mm with necrotic tissue adhered to the superficial layer (Fig. 5A). Histologically, the tumor was grayish-white, dense, and situated in the intrinsic muscularis propria, with atypical spindle-shaped cells showing atypical nuclear fission (Fig. 5B). The colon serosa exhibited fibrous hyperplasia, vessel hyperplasia, inflammatory cell infiltration, and necrotic tissue (Fig. 5C). Immunohistochemical analysis showed that the tumor cells were positive for α1-antichymotrypsin (Fig. 5D), vimentin (Fig. 5E), MDM2, and CDK4, and negative for S-100- and HMB-45. Thus, the possibility of dedifferentiated liposarcoma could not be ruled out. Pathological examination revealed an undifferentiated SCS centered on the anastomosis. The patient recovered well. After 1 year, computed tomography and colonoscopy did not reveal any signs of local recurrence or distant metastasis. Written informed consent was obtained from the patient for the publication of this report and accompanying images.

## 3 Discussion

Sarcomas are malignant mesenchymal tumors subdivided into osteosarcomas and soft-tissue sarcomas. Gastrointestinal sarcomas occur more commonly in the small intestine, stomach, and esophagus of older adults [6]. Common clinical manifestations have been reported to be abdominal pain, abdominal mass, and fever, in that order [7,8]. Undifferentiated sarcoma, a soft-tissue sarcoma first described and named by Ozzello et al. [9], is a high-grade disease with a reported median survival of <6 months [10]. SCS is a type of undifferentiated sarcoma, characterized by the presence of spindle cells and usually originating from the extremities. Although SCSs have been reported to occur intraperitoneally, there are no previous reports of SCSs occurring intracolonally or at the anastomotic site, as was the case here.

As part of the natural response to soft tissue injury, spindle cells present in the injured tissue begin to divide to promote healing. Normally, these cells stop replicating as the affected area heals. However, for reasons not fully understood, they may continue to divide uncontrollably. The cells in excess may then accumulate and combine to form an SCS. Risk factors include Paget's disease of bone, radiation therapy for bone infarction or cancer, and osteomyelitis. We hypothesized that chronic inflammation of the isthmus may have contributed to the observed outcome.

SCSs belong to a group of tumors that are morphologically composed of spindle-shaped cells. Making a diagnosis based on morphology alone is difficult, and a final diagnosis is based on immunostaining and genetic testing. In the present case, the tumor developed at the anastomotic site, and sarcomatoid changes of carcinoma were first considered, but this possibility was ruled out because there was no clear evidence of carcinoma, and the tissue was negative for AE1/AE3. The other immunostaining results showed high malignancy and suggested a high-grade SCS. Although the tumor originated within the intestinal wall, gastrointestinal stromal tumor (GIST) and leiomyosarcoma were also ruled out owing to differences in histology. Synovial sarcoma and clear cell sarcoma were also ruled out, leading to the diagnosis of sarcoma with an unknown differentiation direction.

In contrast, cells were positive for MDM2 and CDK4, suggesting the possibility of a dedifferentiated component in dedifferentiated liposarcoma (DLPS). In such cases, a well-differentiated liposarcoma (WLPS) component is often present in the surrounding areas. In the present case, no WLPS component was found in the excised specimen. There was a preoperative history of fluid retention and spontaneous disappearance of the nodule. Although spontaneous resolution of WLPS has not been previously reported, this is difficult to assess in practice. Only one small nodule was found intraoperatively in the abdominal cavity and was submitted to pathology. However, the diagnosis was scar tissue, and no WLPS component was detected in the resection specimen. Therefore, although the possibility of a DLPS diagnosis is low in this case, careful follow-up will continue because this condition is rare and no clear treatment guidelines have been established.

To the best of our knowledge, this is the first report of SCS occurring in the colon. The frequent recurrence of undifferentiated soft-tissue sarcoma is problematic. Fortunately, the tumor did not show distant metastasis and could be completely resected surgically. However, undifferentiated sarcomas metastasize early, and once distant metastasis develops, it cannot be radically removed by surgery, and the prognosis is very poor. The 2-year overall survival rate for anaplastic sarcoma has been reported to be approximately 60 %. The generally short follow-up period for patients with anaplastic sarcoma, with some patients missing follow-up data and no available reports of survival rates beyond 5 years, indicates that the overall prognosis for patients with anaplastic pleomorphic sarcoma of the colon may be even worse [7]. Local recurrence is also common in soft-tissue sarcomas, within a reported median period of 19 [11] and 15.7 months [12]. Thus, frequent follow-up is recommended after sarcoma resection, especially during the first 2 years [13]. Although complete surgical resection is the mainstay of treatment, there are no fixed standards for lymph node dissection, partly owing to the lack of reports. In the present case, although lymph node dissection was limited to D1 dissection, complete resection was achieved grossly and image-wise, and a certain prognosis was expected. Adjuvant radiation therapy and chemotherapy may be tried postoperatively, depending on the condition and intraoperative status of the patient, but further research is needed to select a specific regimen because of individual differences in efficacy. Adriamycin and ifosfamide should be considered in the treatment of soft-tissue sarcomas if there is suspicion of recurrence. Molecular biology studies of undifferentiated sarcoma of the colon may provide the basis for targeted therapies and immunotherapy, and offer hope for an improvement in the prognosis of these patients.

## 4 Conclusion

SCS is a rare disease, and this is the first reported case occurring in the colon or during colonic anastomosis. The treatment strategy remains controversial, and additional cases are needed to determine the optimal approach.

## Author contribution

NK and MK conceived the presented idea, developed the theory, and performed the computations. MT, MN, TA, MI, RH and KT encouraged the investigation of specific aspects and supervised the findings of this study. All authors discussed the results and contributed to the final manuscript.

## Consent to participate

Written informed consent was obtained from the patient to publish this case report with the accompanying images.

## Ethics approval

This study was approved by the ethics committee of our institution and conducted in accordance with the Declaration of Helsinki.

## Guarantor

Nao Kitasaki

## Funding

This research did not receive any specific grant from funding agencies in the public, commercial, or not-for-profit sectors.

## Conflict of interest statement

None.

## Data Availability

Data sharing is not applicable to this article as no datasets were generated or analyzed during the current study.
